# Enhancing clinician participation in quality improvement training: implementation and impact of an evidence-based initiative to maximise antenatal clinician participation in training regarding women’s alcohol consumption during pregnancy

**DOI:** 10.1186/s12913-022-07717-9

**Published:** 2022-03-26

**Authors:** J. Dray, M. Licata, E. Doherty, B. Tully, B. Williams, S. Curtin, D. White, C. Lecathelinais, S. Ward, S. Hasson, E. J. Elliott, J. Wiggers, M. Kingsland

**Affiliations:** 1 School of Psychology, The University of Newcastle, Callaghan, New South Wales 2308 Australia; 2 School of Medicine and Public Health, The University of Newcastle, Callaghan, New South Wales 2308 Australia; 3 Priority Research Centre for Health Behaviour, University of Newcastle, University Drive, Callaghan, New South Wales 2308 Australia; 4 Hunter New England Population Health, Hunter New England Local Health District, Wallsend, New South Wales 2287 Australia; 5 Hunter Medical Research Institute, New Lambton Heights, New South Wales 2305 Australia; 6 Maternity and Gynaecology, John Hunter Hospital, New Lambton Heights, New South Wales 2305 Australia; 7 Maternity and Gynaecology, Tamworth Hospital, Dean St, Tamworth, New South Wales 2340 Australia; 8 Maternity and Gynaecology, Manning Hospital, York street, Taree, New South Wales 2430 Australia; 9 Foundation for Alcohol Research and Education, Deakin, ACT 2600 Australia; 10 Faculty of Medicine and Health, Discipline of Child and Adolescent Health, University of Sydney, Sydney, New South Wales 2006 Australia; 11 Sydney Children’s Hospital Network, Kids’ Research Institute, Westmead, New South Wales 2145 Australia

**Keywords:** Training, Quality improvement, Maternity, Alcohol

## Abstract

**Background:**

There are significant challenges in ensuring sufficient clinician participation in quality improvement training. Clinician capability has been identified as a barrier to the delivery of evidence-based care. Clinician training is an effective strategy to address this barrier, however, there are significant challenges in ensuring adequate clinician participation in training. This study aimed to assess the extent of participation by antenatal clinicians in evidence-based training to address alcohol consumption during pregnancy, and to assess differences in participation by profession.

**Methods:**

A 7-month training initiative based on six evidence-based principles was implemented in a maternity service in New South Wales, Australia. Descriptive statistics described participation in training (% attending: any training; six evidence-based principles of training; all principles). Regression analyses examined differences by profession.

**Results:**

Almost all antenatal clinicians participated in some training (182/186; 98%); 69% participated in ≥1 h of training (μ = 88.2mins, SD:56.56). The proportion of clinicians participating in training that satisfied each of the six principles ranged from 35% (training from peers and experts) to 82% (training was educational and instructional). Only 7% participated in training that satisfied all principles. A significantly higher proportion of midwifery compared to medical clinicians participated in training satisfying five of the six training principles.

**Conclusions:**

A training initiative based on evidence-based principles resulted in almost all clinicians receiving some training and 69% participating in at least 1 h of training. Variability between professions suggests training needs to be tailored to such groups. Further research is required to determine possible associations with care delivery outcomes.

**Trial registration:**

Australian and New Zealand Clinical Trials Registry, No. ACTRN12617000882325 (date registered: 16/06/2017).

## Background

Internationally, clinical practice guidelines recommend the implementation of antenatal care addressing alcohol consumption by pregnant women [1, 2]. However, research from countries including Canada [3], the United Kingdom [4] and Australia [5–10] indicates provision of such care is limited, including lack of routine assessment of alcohol consumption by pregnant women [5–7] and inconsistent provision of advice on potential harms of such consumption [3–6].

Clinician capability has been identified as a significant barrier to clinicians routinely providing recommended best practice care [11]. In Australia and internationally, studies have found lack of knowledge [12], skill [7, 10, 13] and confidence [10, 11] to be significant barriers to the provision of care according to care delivery guidelines generally [14], as well as a barrier to clinicians providing recommended antenatal care addressing alcohol consumption during pregnancy [7, 11, 12].

Clinician training has been identified in multiple systematic reviews as a key intervention strategy for addressing clinician barriers related to the implementation of evidence-based care [15, 16]. A Cochrane systematic review of 81 trials including over 11,000 health professionals found increased compliance with recommended clinical practice guidelines (improvements in compliance from 2.9 to 15.3%, median of 6%) following educational meetings [15]. A further Cochrane systematic review that included 69 studies involving more than 15,000 health professionals in educational outreach visits (onsite education/training) found that such training increased compliance with desired practice by 5.6% (risk difference; interquartile range 3.0 to 9.0%) [16].

Significant challenges have been reported in ensuring all targeted clinicians participate in training [17–19]. Reviews have reported an average of only 53% of targeted clinicians participate in training in clinical settings generally [15] and 15–75% of clinicians in maternity service settings [17, 20, 21]. Reviews have reported that factors contributing to less than optimal training participation including organisational constraints (e.g. available resources, time) [18, 19, 22], lack of tailoring to local context and different users [21], lack of integration of training into routine organisational meetings and opportunities [18], and lack of organisational support for training to occur [22]. To address these barriers and maximise the effectiveness of training, Cochrane and other systematic reviews recommend that clinician training initiatives address the following six principles: varied structure (one-on-one and group) [22]; multiple modes (online and face-to-face) [23]; multiple formats (interactive and didactic) [15]; mixed content (educational and instructional) [15, 20, 23, 24]; varied facilitators (peers and experts) [16, 25]; and total training of ≥1 h [22]. Participating in training based on these principles individually has been shown to significantly improve practice outcomes, including improvements in care [16, 22, 25–28], increased patient-centred skills [20], and positive effect on patient health behaviours and health status [20]. However, no studies could be located of training initiatives that included all six evidence-based principles, nor any studies of clinician participation in such a training initiative.

We could locate a few studies that incorporated some, but not all of the recommended principles in the design of training programs. For instance, a study in general practice in Australia covered four of six of these evidence-based principles. This study included a weekly group-based training sessions with duration ≥1 h (2.5 h per session, for 6 weeks) that had multiple formats (didactic and interactive), mixed content (educational and instructional) and varied facilitators (peers and experts). This training resulted in 41% of the General Practitioners (*N* = 108) that had self-selected into the training receiving all components of the training program (totalling 15 h of training) [29]. The study did not report receipt of the incorporated evidence-based principles of training. A further study, also in general practices in Australia, qualitatively evaluated a multi-component training program that included content that was delivered through multiple modes (online and face-to-face), with mixed content (educational and instructional) and included peer delivery. Fifteen general practitioners self-selected into the training program, however the percentage of practitioners who participated in the training was not reported, nor was receipt of the incorporated evidence-based principles of training [30]. The ability to achieve sufficient clinician participation in a training initiative implementing all six evidence-based principles of training has not been reported.

The aims of this study were to assess the extent of participation by antenatal clinicians in evidence-based training on care for women for alcohol consumption during pregnancy, and to assess any differences in participation by profession.

## Methods

### Study design

A post-test evaluation of a training program was undertaken utilising data collected during implementation. The study was undertaken as part of a trial to assess the effectiveness of a multi-strategy practice change intervention in increasing clinician delivery of antenatal care addressing women’s alcohol consumption [31]. Clinician training was one of seven practice change strategies that formed the intervention.

### Study setting

The study was conducted in a large metropolitan maternity service in New South Wales, Australia that provides care to over 4000 women annually [32].

### Ethics

Ethics approval was obtained from HNELHD Human Research Ethics Committee (no. 16/10/19/5.15), The University of Newcastle Human Research Ethics Committee (no. H-2016-0422) and Aboriginal Health and Medical Research Council (1236/16).

### Participants

Eligible participants were all clinicians within the participating maternity service who provided antenatal care between February and August 2018. Participants included Clinical Midwife Educators, registered midwives, midwifery students, Aboriginal Health Workers, and medical staff (including Consultants, Registrars; Resident Medical Officers (RMOs), and; general practitioners attending the service as visiting medical officers). To identify eligible staff, employee information was obtained from the organisation’s learning and development unit, and rostering and payroll information was obtained from maternity services. For the length of the training initiative, checks were undertaken of the above information sources each fortnight to identify new eligible staff. Various methods were used to offer and promote training components to staff across clinical professions, including: verbal or written (i.e. email) invitations from key antenatal staff (e.g. Midwifery Unit Managers, Clinical Midwife Educators, Medical Education Fellows, and Senior Medical Consultants), and visual promotional materials (e.g. posters detailing what training sessions were being held, when and where) in common areas such as staff tea rooms, meeting rooms or hospital-based lecture rooms. All eligible staff were invited and encouraged to attend any and all components of the training program.

### Training initiative

A seven-month training initiative was implemented between January and August 2018.

Four steps were undertaken to develop the initiative.

#### Step 1. Review of principles of training

Findings from Cochrane and other systematic reviews were examined to identify recommended principles of effective clinical training initiatives (see Table 1) [15, 16, 20–28, 33, 34]. A multi-layered approach to training was developed, with the intention of addressing all recommended training principles in the training initiative across multiple training components.

**Table 1:** Summary of findings from systematic reviews regarding recommended principles of effective health professional training initiatives

Principle	Description of principle	Evidence
**Mixed content** (educational and instructional)	• Include educational content on serious patient outcomes • Include instructional content on use of Electronic Health Information (EHI)	• Improved training attendance when content/outcomes are perceived as serious [15, 20] • Improved effects if clinicians are provided with an EHI platform and trained in use of the platform [23]
**Adequate total duration** (total minutes received)	• Duration of at least 1 h	• Improved outcomes with total training duration of at least 1 h providing there is adequate follow-up and monitoring of progress [22]
**Varied facilitators** (peers and experts)	• Include sessions conducted by a peer • Include session(s) conducted by an expert	• Slightly improved level of change when educational sessions are conducted by a peer compared to a non-peer [16] • Some support for the use of a local opinion leader/expert opinion in practice change initiatives [25]
**Multiple formats** (interactive and didactic)	• Include a mix of interactive and didactic training	• Improved outcomes with mixed interactive and didactic/lecture-based educational meetings rather than inclusion of only didactic or only interactive sessions [15]
**Multiple modes** (online and face-to-face)	• Include both online and face-to-face training	• Insufficient evidence to support online learning only [23]
**Varied structures** (one-on-one and group)	• Include a mix of groupings	• Significant effects for both one-on-one or group delivered training [22]

#### Step 2. Consultations with Maternity Services and review of existing maternity training systems and opportunities

Consultations were held with key antenatal staff (e.g. Midwifery Unit Managers, Clinical Midwife Educators, Medical Education Fellows, and Senior Medical Consultants). Consultations involved: discussion of potential facilitators of training (e.g. peers, experts), a review of existing training systems (e.g. mandatory full training days, staff online health learning platforms, roles of staff in existing educator positions), and identification of suitable times and locations to implement the training with antenatal clinicians within existing routine training and clinical procedures and opportunities. This included consideration of different clinical professions and service types (hospital-based clinician, outreach clinics, and Aboriginal Maternal and Infant Health Service (AMIHS).

#### Step 3. Embedding cultural safety and inclusion across all training areas

Consultation with and participation of Aboriginal women, community members, Aboriginal Maternal Infant Health Service (AMIHS) staff, and Aboriginal health district staff was led by Aboriginal team members to ensure the lived experience and perspective of Aboriginal women was embedded in the training. This included training addressing: the creation of culturally safe clinical environments; all women (Aboriginal and non-Aboriginal) being asked about their alcohol consumption at multiple time points as a component of routine antenatal care; sensitive and open conversation styles; understanding when to apply clinical discretion; referrals to Aboriginal culturally appropriate support services; clinician competence and confidence in referring Aboriginal women to such support services, and knowledge and use of culturally appropriate resources.

A Clinical Midwife Educator (CME) was appointed full time to facilitate the face-to-face training components for the 7-month training initiative, including on-site training support in antenatal clinics.

#### Step 4. Refinement of the training program

Information obtained through steps 1 to 3 were synthesised: evidence based principles of training (Step 1) were matched to training opportunities and trainers identified during consultations with Maternity Services (Step 2). Information obtained from consultation with and participation of Aboriginal women, community members, Aboriginal Maternal Infant Health Service (AMIHS) staff, and Aboriginal health district staff (Step 3) informed key content requirements for each training component. The resulting training program is detailed in Table 2.

**Table 2:** Summary of the training program

Program component/session	Content (educational/instructional)	Facilitator (Peer/Expert)	Format (Interactive/didactic)	Structure (One-one-one/group)	Mode (face-to-face/online)	Duration (mins)
**Online training module ‘Antenatal Care for Alcohol Consumption during Pregnancy’**	Developed through HETI and adapted from the Women Want to Know project content developed by the Foundation for Alcohol Research and Education (FARE) in collaboration with leading health professionals across Australia including the College of Midwives and the Royal Australian and New Zealand College of Obstetricians and Gynaecologists. Educational: - Serious outcomes, effects/harms of alcohol consumption during pregnancy. - All elements of care (assess, advise, refer). - Myths, beliefs, assumptions - Skills for effective and/or sensitive conversations. Instructional: - Fitting in to practice: encouraging adoption of evidence-based guidelines. - Using eMaternity (e.g., navigation, completion of AUDIT-C).	NA	Interactive	One-on-one	Online	~ 45 mins
**Overview presentation**	Educational: - Serious outcomes, effects/harms of alcohol consumption during pregnancy. - All elements of care (assess, advise, refer). Instructional: - Fitting in to practice: encouraging adoption of evidence-based guidelines. - Using eMaternity (e.g. navigation, completion of AUDIT-C).	Peer (CME)	Didactive	Group	Face-to-face	20–30 min
**Expert presentation**	Educational: - Serious outcomes, effects/harms of alcohol consumption during pregnancy including less well known consequences (i.e. those in addition to FASD e.g. low birth weight, stillbirth, later effects in childhood and into adulthood etc.). - All elements of care (assess, advise, refer). - Myths, beliefs, assumptions.	Expert [35]	Didactive Educational material: 2 page summary of presentation produced and distributed to clinicians (educational materials)	Group	Face-to-face	60 mins
**Case study session**	Educational: - All elements of care (assess, advise, refer). Instructional: - Fitting in to practice: encouraging adoption of evidence-based guidelines.	Peer (CME)	Interactive Educational materials: Case study handouts distributed to clinicians	Group	Face-to-face	15–20 min
**One-on-one support from an assigned clinical midwife educator when in the antenatal clinic(s)**	The following content could be covered at any time in this session: Educational: - Serious outcomes, effects/harms of alcohol consumption during pregnancy - All elements of care (assess, advise, refer) Instructional: - Fitting in to practice: encouraging adoption of evidence-based guidelines. - Using eMaternity (e.g. navigation, completion of AUDIT-C). - Resources for use with women distributed: Foundation for Alcohol Research and Education (FARE) women’s brochure [36], Stay Strong and Healthy Postcard [37], and NSW Standard Drinks Charts.	Peer (CME)	Interactive	One-on-one	Face-to-face	5–20 min
**Final summary presentation (to conclude intensive support period)**	Educational: - All elements of care (assess, advise, refer). Instructional: - Fitting in to practice: encouraging adoption of evidence-based guidelines.	Peer (CME)	Didactic	Group	Face-to-face	30–45 min

### Data collection instruments and procedures

A training participation database was developed utilising REDCap [38]. The database was used to record details of eligible clinicians, the components of training they attended, and their receipt of the recommended principles of training. Clinician training participation data were entered into the database by a research team member, for face-to-face sessions this was based on paper-based records of attendance self-completed by clinicians and validated by the facilitator or team leader in attendance on completion of each training session. For online training sessions, completion records were downloaded from the online platform and entered into the database by a research team member on a weekly basis.

### Data collection measures

#### Clinician participation in training

Information regarding training participation recorded in the database included: training session completed/attended, date of completion, training structure (one-on-one, group), mode (online, face-to-face), format (interactive, didactic), content (educational, instructional), facilitator (peer, expert) (all yes/no) and duration. These data were used to report on the following eight training participation outcomes.

Proportion of staff attending:

1. any training
2. training that was mixed content (educational and instructional)
3. training of adequate total duration (≥ 1 h)
4. training with varied facilitators (peers and experts)
5. training in multiple formats (interactive and didactic)
6. training through multiple modes (online and face-to-face)
7. training of varies structures (one-on-one and group)
8. training that satisfied all of the above six principles of training.

### Clinician characteristics

Clinician characteristics data were extracted from records obtained from roster and payroll data systems. Data were extracted on the following characteristics: clinician position, clinical team and service type (core hospital-based clinician, outreach clinics, and AMIHS).

### Statistical analysis

All analyses were conducted using SAS (Version 9.3). Clinician position data were used to create three clinician profession groups (midwifery, medical and Aboriginal Health Workers).

Variables were created to determine the receipt of each principle: ‘both one-on-one and group mode’ (Yes/No); ‘both interactive and didactic format’ (Yes/No); ‘both face-to-face and online type’ (Yes/No); ‘educational and instructional content’ (Yes/No); training obtained > 1 h (Yes/No); and, ‘all principles of training’ (Yes for all).

Descriptive statistics were used to examine clinician characteristics and clinician participation in training. Exact logistic regression was used to investigate associations between clinician participation for each of the six principle of training and ‘all principles of training’ and clinician profession group (midwifery, medical and Aboriginal Health Workers).

## Results

### Clinician sample and characteristics

One-hundred and eighty-six antenatal clinicians were identified as eligible for training. As shown in Table 3, approximately two thirds (67.20%) were midwifes, just over a quarter (27.42%) medical staff and 5.38% Aboriginal Health Workers.

**Table 3:** Descriptive characteristics of clinicians and service types within the priority cohort (*N* = 186)

Characteristic	n (%)
Clinician profession groups and positions	
*Midwifery*	125 (67.20%)
Clinical Midwife Educator	13 (6.99%)
Clinical Midwife Specialist/Consultant	29 (15.60%)
Clinical Nurse Consultant	2 (1.08%)
Managers	8 (4.30%)
Registered Midwife	72 (38.17%)
Student Midwife	3 (1.61%)
*Aboriginal Health Workers*	10 (5.38%)
*Medical*	51 (27.42%)
Consultant	4 (2.15%)
Fellow	2 (1.08%)
General Practitioner	3 (1.61%)
Medical Officer	20 (10.75%)
Registrar	12 (6.45%)
Staff specialist	8 (4.30%)
Service type	
Central hospital-based clinic	138 (74.19%)
Outreach clinics	29 (15.60%)
Aboriginal Maternal and Infant Health Service (AMIHS)	19 (10.21%)

Ninety eight percent (*n* = 182) of the eligible clinicians participated in some training (μ: 88.2 mins/clinician, SD: 56.56 mins/clinician; M: 75 mins/clinician; Min: 0 mins; Max: 278 mins), with 68.8% participating in ≥1 h. The proportion of clinicians participating in training that satisfied each training principle varied between 35% (received training from varied facilitators – i.e., from both peers and expert facilitators) and 82% (received training that mixed content – i.e., was both educational and instructional in content). Only 7% participated in training that satisfied all principles of training (Table 4).

**Table 4:** Clinician participation in training that satisfied principles of training

Principles of training	n (%)
Varied structures	
One-on-one training	88 (47.31%)
Group training	157 (84.41%)
**Both one-on-one and group**	**69 (37.10%)**
Multiple modes	
Online training	84 (45.16%)
Face-to-face training	176 (94.62%)
**Both online and face-to-face**	**78 (41.94%)**
Multiple formats	
Interactive training	142 (76.34%)
Didactic training	147 (79.03%)
**Both interactive and didactic**	**108 (58.06%)**
Mixed content	
Educational	181 (97.31%)
Instructional	154 (82.8%)
**Both educational & instructional**	**153 (82.26%)**
Varied Facilitators	
Peer facilitators	173 (98.3%)
Expert facilitator	64 (36.36%)
**Both peer and expert**	**61 (34.66%)**
Training duration	
< 1 h	58 (31.18%)
**≥ 1 h**	**128 (68.82%)**
Receipt of principles of training	
Received no principles	18 (9.68%)
One principle	25 (13.44%)
Two principles	16 (8.60%)
Three principles	36 (19.35%)
Four principles	29 (15.59%)
Five principles	49 (26.34%)
**All six principles**	**13 (6.99%)**

### Association between clinician participation in training that satisfied training principles and clinician profession group

Significant differences in clinician participation in training that satisfied the training principles were found by clinician profession group. A higher proportion of midwifery compared to medical clinicians received the following principles of training: multiple modes; multiple formats; ≥1-h duration of training; both elements, and; all principles of training. See Table 5.

**Table 5:** Clinician participation in training that satisfied training principles, by clinician profession group

	Midwifery ***N*** = 125	Medical ***N*** = 51	Aboriginal Health Workers (AHW) ***N*** = 10	Regression Analysis		
Principle of training	n (%)	n (%)	n (%)	Medical vs. Midwifery OR [CI] (***p)***	AHW vs. Midwifery OR [CI] (***p)***	***p***
Varied structures						
One-on-one	50 (40.00%)	34 (66.67%)	4 (40.00%)	OR: 2.98 [1.44; 6.35] (*p* = 0.002)	OR: 1.00 [0.20; 4.46] (*p* = 1.00)	0.005
Group	108 (86.40%)	39 (76.47%)	10 (100.00%)	OR: 0.51 [0.21; 1.29] (*p* = 0.17)	OR: 2.13 [0.41; ∞] (*p* = 0.49)	0.12
**Both one-on-one and group**	**42 (33.60%)**	**23 (45.10%)**	**4 (40.00%)**	**OR: 1.62 [0.79; 3.32] (*p*** **= 0.21)**	**OR: 1.31 [0.26; 5.89] (*p*** **= 0.92)**	**0.35**
Multiple modes						
Online	77 (61.60%)	2 (3.92%)	5 (50.00%)	OR: 0.03 [0.00; 0.11] (*p* = < 0.001)	OR: 0.63 [0.14; 2.87] (*p* = 0.69)	< 0.001
Face-to-face	116 (92.80%)	50 (98.04%)	10 (100.00%)	OR: 3.86 [0.51; 173.38] (*p* = 0.32)	OR: 1.03 [0.19; ∞] (*p* = 0.98)	0.41
**Both online and face-to-face**	**72 (57.60%)**	**1 (1.96%)**	**5 (50.00%)**	**OR: 0.01 [0.00; 0.09]** **(*p*** **= < 0.001)**	**OR: 0.74 [0.16; 3.38] (*p*** **= 0.88)**	**< 0.001**
Multiple formats						
Interactive	99 (79.20%)	36 (70.59%)	7 (70.00%)	OR: 0.63 [0.28; 1.44] (*p* = 0.30)	OR: 0.62 [0.13; 3.94] (*p* = 0.73)	0.37
Didactic	102 (81.60%)	36 (70.59%)	9 (90.00%)	OR: 0.54 [0.24; 1.25] (*p* = 0.16)	OR: 2.02 [0.26; 92.76] (*p* = 0.88)	0.18
**Both interactive and didactic**	**81 (64.80%)**	**21 (41.18%)**	**6 (60.00%)**	**OR: 0.38 [0.18; 0.78] (*p*** **= 0.007)**	**OR: 0.82 [0.18; 4.15] (*p*** **= 1.00)**	**0.016**
Mixed content						
Educational (e.g. serious outcomes, effects/harms of alcohol consumption during pregnancy	117 (93.60%)	37 (72.55%)	10 (100.00%)	OR: 0.18 [0.06; 0.51] (*p* = < 0.001)	OR: 0.91 [0.16; ∞]] (*p* = 1.00)	< 0.001
Instructional (e.g. using eMaternity including navigation and completion of AUDIT C)	117 (93.60%)	46 (90.20%)	10 (100.00%)	OR: 0.63 [0.17; 2.58] (*p* = 0.62)	OR: 0.91 [0.16; ∞] (*p* = 1.00)	0.59
**All content**	**116 (92.80%)**	**34 (66.67%)**	**10 (100.00%)**	**OR: 0.16 [0.06; 0.41] (*p*** **= < 0.001)**	**OR: 1.03 [0.91; ∞] (*p*** **= 0.98)**	**< 0.001**
Varied facilitators						
Peer	115 (99.14%)	48 (96.00%)	10 (100.00%)	OR: 0.21 [0.00;4.14] (*p* = 0.43)	OR: 0.09 [0.00;∞] (*p* = 1.00)	0.34
Expert	41 (35.34%)	18 (36.00%)	5 (50.00%)	OR: 1.03 [0.48;2.16] (*p* = 1.00)	OR: 1.82 [0.39;8.41] (*p* = 0.55)	0.69
**Both peer and expert**	**40 (34.48%)**	**16 (32.00%)**	**5 (50.00%)**	**OR: 0.89 [0.41;1.91] (*p*** **= 0.90)**	**OR: 1.89 [0.41;8.74] (*p*** **= 0.51)**	**0.55**
Adequate total duration						
**≥ 1 h**	**94 (75.20%)**	**26 (50.98%)**	**8 (80.00%)**	**OR: 0.35 [0.16;0.72] (*p*** **= 0.004)**	**OR: 1.32 [0.24;13.38] (*p*** **= 1.00)**	**0.006**
**All principles of training**	**12 (9.60%)**	**0 (0.00%)**	**1 (10.00%)**	**OR: 0.13 [0.00; 0.66] (*p*** **= 0.028)**	**OR: 1.05 [0.02; 8.80] (*p*** **= 1.00)**	**0.035**

## Discussion

This study aimed to assess the extent of antenatal clinician participation in a multi-component evidence-based training initiative designed based on six principles of training, and to assess differences in participation by profession. The comprehensive training initiative was effective in achieving substantial levels of clinician participation, with 98% of clinicians participating in some training and approximately two thirds of clinicians (68%) participating in at least 1 h of training across the training components. Few clinicians (7%) received training that satisfied all evidence-based principles of training. The training principles related to content (82%), duration (68%) and format (58%) were able to be satisfied for more than half of antenatal staff, whereas the principles related to mode (42%), structure (37%) and facilitator (35%) were not.

As noted in systematic reviews, data on level of participation in training is not often clearly reported in included studies (44%) [15]. For those studies that have reported training participation, reported participation rates for training with maternity services clinicians range from 15 to 75% [17, 20, 21]. The present study found the proportion of staff to receive any training to be 98%; a result notably high in comparison to these past studies. None of these past studies reported on training participation in terms of any of the principles of training that were reported in this study.

There were a number of factors that may have resulted in different levels of clinician participation in training incorporating each of the principles. In terms of facilitator, 98% of staff received training from a peer, whereas only 36% received training provided by an expert. This may have been due to less opportunities (two face to face group sessions) provided by an expert, compared to the 7-month presence of a Clinical Midwife Educator as a peer facilitator. The use of additional modes (e.g. online or pre-recorded sessions) for delivering expert-delivered training may be needed to be implemented to enable this principle to be satisfied by a greater proportion of staff.

In respect to structure, 84% of staff attended a group session compared to only 47% of staff participating in one-on-one training sessions. This was impacted by logistical challenges in reaching all 186 target staff with one-on-one sessions, including changing rosters, staff rotations, limited dedicated time for such sessions and complexity of opportunistic one-on-one sessions at outreach sites. In contrast, group training sessions, are able to be justifiably attended for training purposes. If the principle of mixed structure (one-on-one and group) is to be satisfied, there needs to be identification of further opportunities for one-on-one training in such clinical settings as well as organisational support and recognition to allow time to be dedicated to such sessions and their role in training legitimised.

In terms of training mode, 95% of staff were able to attend face-to-face training. Such training was able to be provided with greater flexibility in terms of session length and format, which may have assisted with reach. This compared to online training which was only undertaken by 45% of staff. Digital literacy could have contributed and has been identified in existing literature [39]. Also, limited availability of computers within clinical spaces that were able to be utilised to undertake training and limited time allowed to undertake online training were both recognised issues identified by participating clinicians that may have contributed to this result. Additional dedicated computing resources may be required to overcome these barriers and assist in the principle of mixed mode (online and face-to-face) being satisfied.

There were significant differences in receipt of principles of training by clinical profession with a higher proportion of midwifery compared to medical receiving the following principles of training: multiple modes; multiple formats; ≥1-h duration of training; and mixed content. Despite being offered the same training opportunities no medical staff received training that satisfied all training principles. This aligns with barriers to participation in training by medical staff cited in related literature including: organisational constraints (e.g. available resources and organisational support for training to occur) [18, 19, 22], lack of tailoring to different contexts and users [21], and lack of time to participate in training due to increasing workloads, pressures and staff shortages [18]. During consultations held with maternity staff during planning of the training program, consideration was given to potential differences in training needs by clinical profession. It was noted that medical staff required delivery of the training content in short (5–15 min) sessions to fit within existing shifts and training schedules, compared to midwifery staff who had longer blocks of time (30–120 min) available within existing schedules (e.g. mandatory full training days). Despite adapting training to such profession-specific needs, difficulty securing time for training with medical staff was a key barrier in ensuring this profession group received the same duration of training as midwifery staff and therefore exposure to training that satisfied the training principles. This variability of receipt of training suggests further research is needed to better understand the barriers that different professions (particularly medical staff) face in participating in training programs and to test the effectiveness of strategies to overcome such barriers.. To encourage greater uptake of training for medical staff, it is likely that such strategies will need to include protected time for staff to complete training programs and funding to backfill clinical shifts.

The study has a number of strengths including delivery of training as part of routine clinical education, the use of observed training attendance data and coding of training content based on evidence-based principles. The study should also be considered in light of its design and methodological characteristics. The study was undertaken within a single maternity service in a metropolitan hospital. It is unknown how comparable the training opportunities or clinician response in this setting are to other clinical settings without further evaluation across multiple sites. However, the hospital setting in which this trial was conducted was inclusive of varied antenatal models (hospital based, community outreach, AMIHS) and staffing types. The size of the sample was limited to the number of staff within the service and more precision of the findings would have been achieved with a larger sample.

## Conclusions

This comprehensive, multi-component, evidence-based approach to clinician training was effective in achieving substantial clinician participation in training opportunities, and at a level that is greater than previously reported. Variability between professions suggests further tailoring of training is required, particularly for medical staff. Further research is required to determine whether participation in training was able to address the clinician barriers to care delivery that it was designed to address, including knowledge, confidence and skill, and to evaluate the contribution that the training program made to changing clinician practices as a component of a multi-strategy practice change intervention [31]. It is likely well-designed training programs, when delivered together with other practice change strategies, including reminders and audit and feedback, that address other clinician barriers to care delivery will increase levels of recommended care [40].

## Data Availability

The datasets generated during and analysed during the current study are not publically available due to requirements pertaining to confidentiality and privacy of study participants, but are available from the corresponding author on reasonable request.

## Abbreviations

RMOs: Resident Medical Officers
AMIHS: Aboriginal Maternal and Infant Health Service
CME: Clinical Midwife Educator
EHI: Electronic Health Information
AHW: Aboriginal Health Workers
